# Trained immunity: A new player in cancer immunotherapy

**DOI:** 10.7554/eLife.104920

**Published:** 2025-06-18

**Authors:** Shu Li, Yi Zou, Austin McMasters, Fuxiang Chen, Jun Yan

**Affiliations:** 1 Division of Immunotherapy, The Hiram C. Polk, Jr., MD Department of Surgery, Immuno-Oncology Program, Brown Cancer Center, University of Louisville School of Medicine Louisville United States; 2 Department of Clinical Laboratory, Shanghai Ninth People’s Hospital, Shanghai Jiao Tong University School of Medicine Shanghai China; 3 Department of Microbiology and Immunology, University of Louisville School of Medicine Louisville United States; 4 College of Health Science and Technology, Shanghai Jiao Tong University School of Medicine Shanghai China; Radboud University Nijmegen Medical Centre Netherlands; Yale University United States

**Keywords:** trained immunity, innate immune cells, cancer immunotherapy

## Abstract

In the past, immune memory was considered an exclusive feature of the adaptive immune system. However, accumulating evidence suggests that the innate immune system, the most primitive and fundamental component of immunity, can mount more robust responses to non-specific stimuli following prior exposure to different types of initial stimuli, a phenomenon known as trained immunity. Trained immunity has been extensively studied in diverse disease contexts, including infectious diseases, autoimmune disorders, and chronic inflammatory conditions. Notably, significant advancements have been made in recent years in understanding the roles and therapeutic potential of trained immunity in oncology. This review aims to explore the multifaceted roles of trained immunity across different cancer types, providing a comprehensive summary of the pertinent stimuli and associated molecular mechanisms. Additionally, we evaluate the clinical applications of various trained immunity stimuli in cancer therapy and offer perspectives on future directions for integrating trained immunity into cancer treatment strategies.

## Introduction

**Figure 1.**
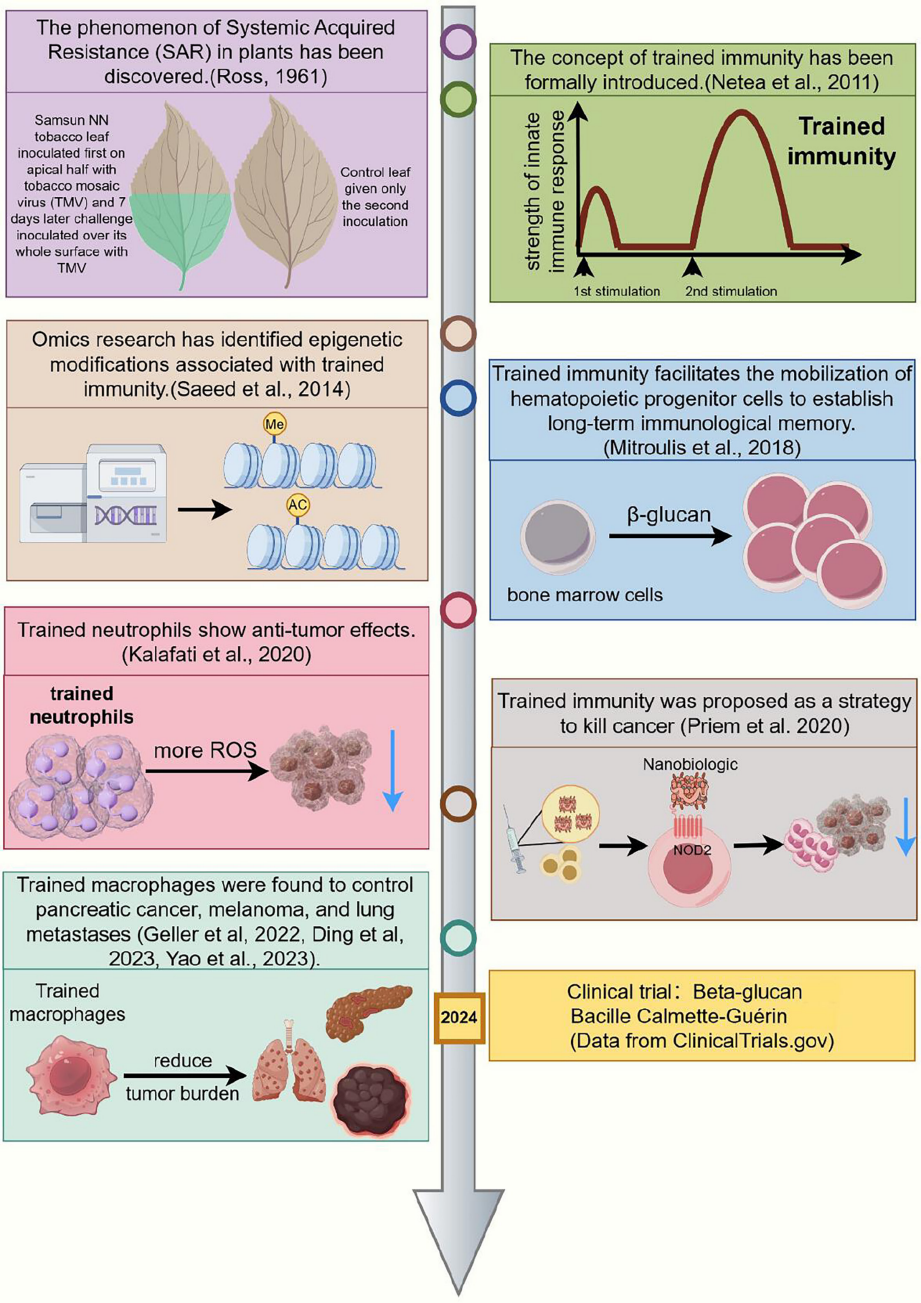
Chronology of significant milestones in research on trained immunity and cancer treatment. This timeline highlights major developments in trained immunity from 1961 to 2024. The concept originated with the discovery of Systemic Acquired Resistance (SAR) in plants (118) and was formally introduced as ‘trained immunity’ in 2011 (Netea et al., 2011). In 2014, omics studies identified key epigenetic modifications associated with trained immunity (Saeed et al., 2014). By 2018, research demonstrated that trained immunity could mobilize hematopoietic progenitors to establish long-term innate immune memory (Mitroulis et al., 2018). In 2020, trained neutrophils were shown to exhibit antitumor effects (Kalafati et al., 2020), and trained immunity was proposed as a strategy for cancer treatment (Kalafati et al., 2020; Priem et al., 2020). Subsequently, trained macrophages were found to control pancreatic cancer, melanoma, and lung metastases (Geller et al., 2022; Wang et al., 2023; Ding et al., 2023). Most recently, in 2024, clinical trials have explored the use of β-glucan and Bacillus Calmette-Guerin (BCG) vaccines to induce trained immunity (ClinicalTrials.gov). This figure illustrates the evolution of trained immunity research and its potential applications in cancer therapy. This figure was created using FigDraw.

Since Edward Jenner laid the foundation for immune memory through the successful application of the cowpox vaccine in the late 18th century, immune memory was long thought to be the exclusive domain of adaptive immunity. For hundreds of years, this understanding remained unchallenged. However, growing evidence has revealed that innate immunity – the most evolutionarily conserved and fundamental defending system – also possesses a unique form of immune memory. To describe this concept, the term *trained immunity* was formally introduced in 2011 (Netea et al., 2011), marking a significant shift in our understanding of the immune system (Figure 1).

Similar to adaptive immunity, trained immunity refers to the altered responses of innate immune cells to stimuli based on prior exposures. However, these secondary responses may vary: they can be either enhanced or weakened, leading to some confusion about the concept of ‘training’. To address this, in 2016, the term *trained immunity* was refined to specifically describe the scenarios where innate cells exhibit enhanced immunity following initial exposure. In contrast, cases where innate immune responses are weakened were categorized as *tolerized innate immunity* (Netea et al., 2016).

Although both forms of immunity share the concept of immune memory, innate immunity exhibits this memory in a way that is significantly distinct from adaptive immunity. The most striking feature of trained immunity is the broad and rapid responses of trained innate immune cells. Unlike adaptive immune cells, which rely on specific antigen recognition receptors such as T cell receptor and B cell receptor, innate immune cells respond independently of the strict ‘key & lock’ antigen recognition pattern. Instead, they employed pattern recognition receptors to detect pathogen-associated molecular patterns (PAMPs) or damage-associated molecular patterns (DAMPs) (Medzhitov and Janeway, 2002). This ‘non-specific’ training enables innate immune cells to provide broad and robust protection against a wide range of stimuli. Additionally, due to this mode of stimulus recognition, the complex immunological processes typical of adaptive immunity – such as antigen uptake, processing, and presentation by antigen-presenting cells (APCs) – are not required. As a result, trained innate immune cells can mount secondary enhanced responses much more rapidly than immunized adaptive immune cells (Iwasaki and Medzhitov, 2015).

Cancer remains one of the most life-threatening diseases today. According to the International Agency for Research on Cancer (IARC) 2024 report, nearly 20 million people were diagnosed with cancer globally in 2022 (Bray et al., 2024). Despite widespread use of surgical resection, chemotherapy, radiotherapy, or combined treatments, approximately 9.7 million people worldwide died from cancer that same year (Bray et al., 2024). For many patients, especially those with unresectable tumors, the long-term efficacy of traditional chemo- and/or radiotherapy remains unsatisfactory. In recent years, immunotherapy, particularly immune checkpoint inhibitors (ICIs), has significantly improved survival rates in various malignant tumors, especially non-small cell lung cancer (NSCLC) and melanoma (Sharma et al., 2023). This progress highlights an encouraging direction for cancer treatment strategies. However, certain cancers, such as pancreatic ductal adenocarcinoma, respond poorly to immunotherapy. Even for NSCLC and melanoma, the overall objective response rates (ORR) to ICIs remain below 40% (Leinwand and Miller, 2020). Therefore, there is an urgent need to develop novel immunotherapies or enhance the effectiveness of existing immune-oncology treatments.

Given the broad, profound, and rapid effects of trained immunity, its non-specific protection makes it a promising strategy to enhance existing immunotherapies or other antitumor treatments. Several research groups have begun exploring the potential of trained immunity in cancer therapy, with increasingly encouraging results emerging (Figure 1). In this review, we summarize recent advances in the field of trained immunity and cancer treatment and offer our perspectives on future directions for this line of investigation.

### Mechanisms of trained immunity in cancer

**Figure 2.**
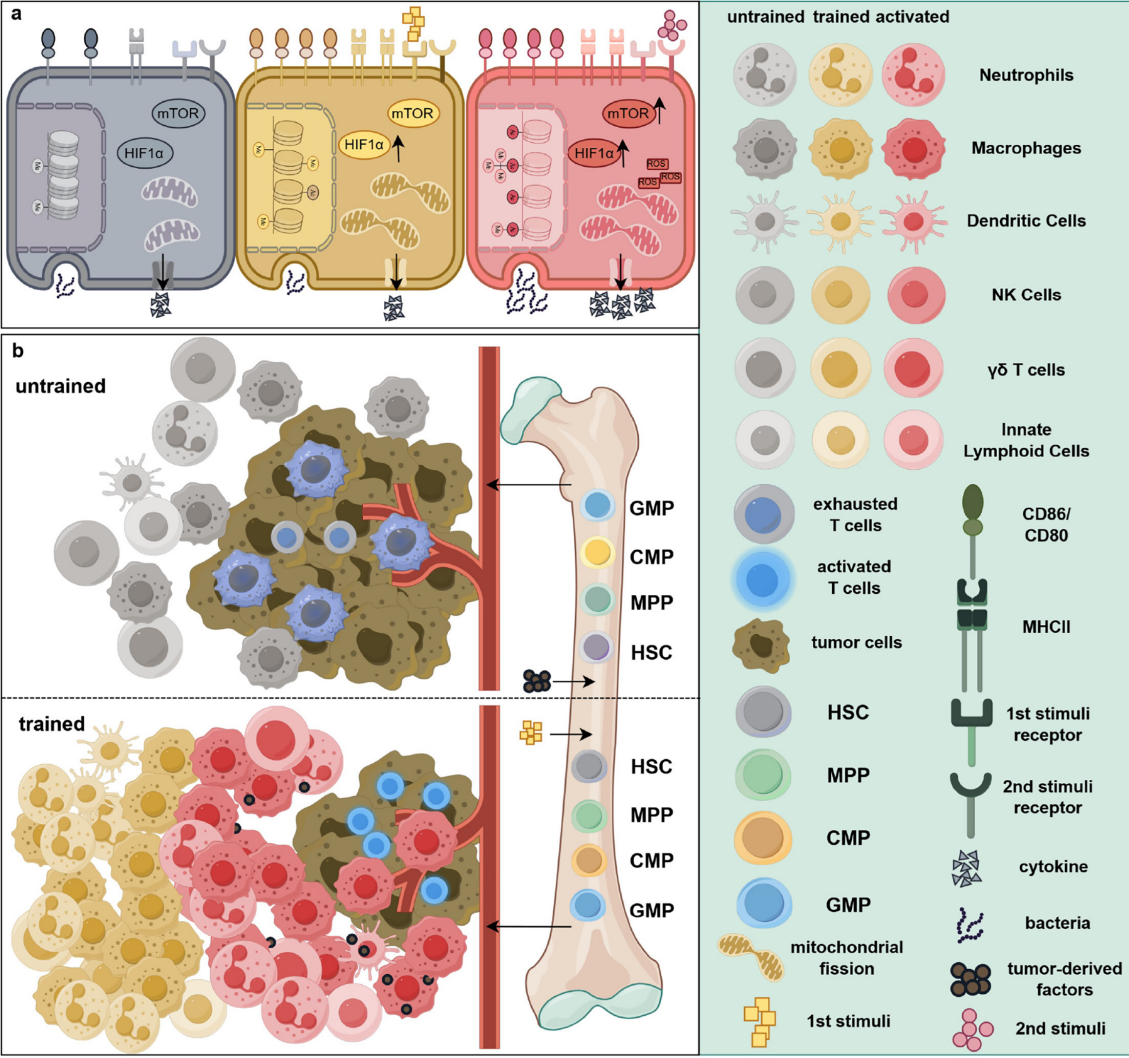
Epigenetic and metabolic reprogramming associated with trained immunity in cancer treatment. (**a**) Intracellular pathway changes associated with trained immunity. Compared to resting cells (gray), immune cells exposed to an initial stimulus exhibit augmented epigenetic modifications, elevated expression levels of mTOR and HIF1α, and increased mitochondrial fission (yellow). In addition, surface markers such as CD80, CD86, and MHC II are upregulated, indicating a pre-activated state. Notably, at this stage, the cells’ ability to secrete cytokines and phagocytose bacteria remains unaltered. Upon exposure to a secondary stimulus, these cells (red) transition into a fully activated state, characterized by increased production of reactive oxygen species (ROS) and cytokines, thereby enhancing their tumoricidal efficacy. (**b**) The differential effects of tumor-associated factors on hematopoiesis. In the absence of trained immunity-inducing stimuli, tumor-derived factors promote the differentiation of immunosuppressive myeloid cells including neutrophils and macrophages from bone marrow progenitors. These cells infiltrate the tumor microenvironment, leading to immune cell suppression, exhaustion, or dormancy. In contrast, exposure to trained immunity-inducing agents induces myelopoiesis, facilitating the mobilization of a greater number of trained monocytes and/or neutrophils into the peripheral circulation. Consequently, more activated immune cells accumulate within and around the tumor, collectively suppressing tumor progression and metastasis. Abbreviations: CMP, common myeloid progenitors; GMP, granulocyte–macrophage progenitors; HIF1α, hypoxia-inducible factor 1 alpha; HSC, hematopoietic stem cells; mTOR, mammalian target of rapamycin; MPP, multipotent progenitors. This figure was created using FigDraw.

The mechanisms of trained immunity in cancer involve broad shifts in progenitor cell and innate immune cell epigenetics, transcriptional activity, and metabolism (Figure 2). When exposed to training agents such as β-glucan, Bacillus Calmette-Guerin (BCG) vaccination, or trained immunity-modulating nanotherapeutics, hematopoietic stem and progenitor cells (HSPCs), as well as various innate immune cell types, including neutrophils, macrophages, and NK cells, undergo extensive epigenetic, metabolic, and transcriptional reprogramming (Arts et al., 2016b; Arts et al., 2016a; Bekkering et al., 2018; Christ et al., 2018; Kaufmann et al., 2018; Domínguez-Andrés et al., 2019b; Cheng et al., 2014; Saeed et al., 2014; Ding et al., 2023; Geller et al., 2022), leading to both central and peripheral trained immunity. These changes collectively promote the increased transcription of glycolytic genes, enhanced glucose uptake, rapid ATP and lactate production, elevated cytokine production, and heightened immune cell effector functions – processes essential for the induction and maintenance of trained immunity and its therapeutic effects in cancer. In the following section, we discuss the cellular and molecular mechanisms utilized by different training agents and trained innate immune cells to control primary tumor progression and metastasis.

### Central trained immunity in cancer

Central trained immunity pertains to the enduring reprogramming of HSPCs within the bone marrow. This reprogramming significantly influences the differentiation of myeloid cells, predisposing them toward a pro-inflammatory phenotype that can profoundly impact the tumor microenvironment (TME) and cancer progression. Emerging evidence suggests that alterations in the bone marrow niche, along with epigenetic, metabolic, and inflammatory conditions, play crucial roles in shaping the immune landscape in cancer.

One of the most well-studied trained immunity inducers, β-glucan, has been shown to reprogram HSPCs in the bone marrow, leading to the generation of trained neutrophils and macrophages that effectively control primary tumor growth and lung cancer metastasis (Ding et al., 2023; Kalafati et al., 2020). Similarly, BCG vaccine, long used in the treatment of bladder cancer, is believed to exert its antitumor effects in part through the induction of trained immunity (van Puffelen et al., 2020). Notably, intravenous BCG injection reprograms HSPCs and enhances myelopoiesis (Kaufmann et al., 2018). Interestingly, intravenous BCG treatment has resulted in increased CD8+ T cells and reduced myeloid-derived suppressor cells (MDSCs), while intravesical BCG, although FDA-approved for high-risk non-muscle-invasive bladder cancer, has been shown to promote tumor-support TME (Atallah et al., 2024). In contrast, a recent study reported that intra-bladder BCG administration reprograms HSPCs in both mice and humans, leading to increased myelopoiesis and enhanced APC function in myeloid cells, thereby inducing antitumor immunity (Daman et al., 2024). These seemingly contradictory findings underscore the need for further investigation to reconcile route-dependent differences in BCG-mediated immune reprogramming.

Additional studies by Priem et al. demonstrated that the nanobiologic therapeutic agent MTP-HDL reprograms bone marrow progenitor cells, inducing trained immunity that suppresses tumor growth, modulates the TME, and potentiates ICI therapy in murine models of melanoma (Priem et al., 2020). Conversely, obesity-induced metabolic and epigenetic changes in bone marrow progenitors have been shown to skew hematopoiesis toward a myeloid-biased profile, thereby promoting chronic inflammation and potentially facilitating tumor (Xu et al., 2022). Understanding these mechanisms may inform strategies to direct immune training toward enhancing antitumor responses while minimizing tumor-promoting inflammation. Given the strong link between metabolism and cancer, studying how bone marrow metabolic reprogramming influences immune cell development and differentiation may hold a key for overcoming cancer-related immune dysfunction. Notably, Domínguez-Andrés et al. explored the role of the itaconate metabolic pathway in regulating tolerance and trained immunity, suggesting that targeting metabolic pathways in HSPCs could modulate trained immunity and impact cancer immune responses (Domínguez-Andrés et al., 2019b; Domínguez-Andrés et al., 2019a).

Together, these studies highlight the therapeutic potential of long-term innate immune cell reprogramming in cancer therapy. A deeper understanding of the duration, specificity, and modulating factors, including diet, microbiome, and systemic metabolites, affecting bone marrow training may pave the way for innovative strategies in cancer treatment.

### Peripheral trained immunity in cancer

Peripheral trained immunity primarily involves innate immune cells such as monocytes, macrophages, NK cells, and γδ T cells within the circulation or tissues, without involving changes in bone marrow progenitors. The trained innate immune cells mount heightened responses upon re-exposure to pathogens or tumor cells. We previously demonstrated that yeast-derived particulate β-glucan induces peripheral trained immunity in the pancreas, thereby limiting the progression and metastasis of pancreatic cancer (Geller et al., 2022; Woeste et al., 2023). In this study, these particulate β-glucans were shown to traffic predominately to the pancreas, where they induced trained immunity in infiltrating macrophages, monocytes, and neutrophils. Respiratory viral infection, such as influenza A, also induces peripheral trained immunity in tissue-resident alveolar macrophages (AMs), thereby controlling tumor progression in the lungs (Wang et al., 2023). Notably, these trained AMs do not originate from circulating monocytes, suggesting a form of tissue-specific peripheral trained immunity. Interestingly, a recent study showed that adjuvants such as the immunostimulatory antimicrobial peptide DP7 can stimulate trained immunity, specifically in peripheral splenic myeloid cells, thereby eliciting potent antitumor immunity in mouse tumor models (Zhang et al., 2025). This line of research offers new insights into adjuvant mechanisms of action and the development of more effective cancer vaccines.

The modulation of peripheral trained immunity occurs through diverse epigenetic and metabolic alterations. Subhasinghe et al. demonstrated in chickens that administration of CpG-ODN leads to metabolic shifts toward mitochondrial respiration in peripheral immune cells, thereby enhancing their immunoprotective capacity against infections (Subhasinghe et al., 2024). Although this study did not focus on cancer, it offers valuable insights into harnessing peripheral trained immunity to strengthen immune responses. In support of this approach, the use of macrophage membrane-camouflaged BCG vaccine, which specifically targets tumor-associated macrophages for the induction of trained immunity, has been shown to effectively stimulate antitumor responses and control lung cancer development (Zhang et al., 2024).

### Effector trained innate immune cells in cancer

#### Neutrophils

Trained neutrophils induced by β-glucan exhibit enhanced degranulation and increased production of reactive oxygen species, enabling direct tumor cell killing (Kalafati et al., 2020). In addition, type I IFN signaling has been shown to mediate the antitumor activity of trained neutrophils. These neutrophils also release pro-inflammatory cytokines and chemokines that recruit and activate additional waves of immune cells, including monocytes, macrophages, and adaptive immune cells. This pro-inflammatory signaling in the TME promotes robust antitumor responses and is associated with a metabolic shift toward glycolysis, known as the Warburg effect (Cheng et al., 2014). Two critical pathways driving this metabolic shift are the mechanistic target of rapamycin and hypoxia-inducible factor 1-alpha (Cheng et al., 2014), both of which play significant roles in promoting glycolysis. However, neutrophil infiltration and function can also be hijacked by tumors, leading to immunosuppressive signaling that converts neutrophils into polymorphonuclear myeloid-derived suppressor cells (PMN-MDSCs), which dampen antitumor immunity. Previous studies have shown that orally administered yeast-derived particulate β-glucan treatment reduces tumor burden and decreases the accumulation of PMN-MDSC, while simultaneously inducing oxidative burst and apoptosis in these cells (Albeituni et al., 2016). Nonetheless, whether these effects are directly attributable to trained immunity remains to be fully elucidated.

#### Monocytes/macrophages

Trained monocytes and macrophages also undergo significant histone modifications, including methylation and acetylation, during the induction of trained immunity. Similar to neutrophils, these cells exhibit a Warburg effect upon exposure to training agents, resulting in increased production of pro-inflammatory cytokines such as TNFα, IL-1β, IL-6, and IL-12 in response to PAMPs and DAMPs. This promotes immune cell recruitment, inflammation, and tumor cytotoxicity. In macrophages, mitochondrial fission driven by activation of dynamin-related protein 1 (Drp-1) and the sphingosine-1-phosphate (S1P) pathway enhances phagocytic and cytotoxic capabilities (Ding et al., 2023). This training induces a highly polarized M1-like phenotype, characterized by increased antigen presentation, upregulation of co-stimulatory molecule expression (CD80 and CD86), and robust cytokine production (Ding et al., 2023; Geller et al., 2022). Trained monocytes and macrophages are particularly beneficial in overcoming the immunosuppressive TME, as seen in pancreatic cancer, where a dense stromal environment impedes immune infiltration (Geller et al., 2022; Woeste et al., 2023). These trained cells can effectively penetrate the stroma, directly kill tumor cells, and elicit a more effective immune response, especially when combined with irreversible electroporation (IRE) treatment and ICI anti-PD-1 therapy (Geller et al., 2022; Woeste et al., 2023). In contrast to bone marrow-derived monocytes/macrophages, tissue-resident macrophages such as AMs can also acquire trained immunity following influenza A infection as noted above. These trained AMs exhibit potent antitumor functions in the lungs through enhanced phagocytic activity and tumoricidal capacity (Wang et al., 2023). Their antitumor effects depend on NK cells and IFN-γ but are notably independent of T cell help, which contrasts with adenoviral infection-induced trained AMs (Yao et al., 2018), suggesting that different training inducers may imprint distinct functional properties even on the same type of trained cells.

#### NK cells

NK cells play a crucial role in the innate immune response to cancer by targeting tumor cells that have downregulated MHC class I expression. NK cells trained with cytokines such as IL-12, IL-15, and IL-18 or through BCG vaccination demonstrate enhanced production of pro-inflammatory cytokines, particularly IFN-γ, making them ideal partners for CD8+ T cells in generating a robust antitumor response (Kleinnijenhuis et al., 2014; Tarannum and Romee, 2021). In the context of cancer, NK cells can also support the induction of trained immunity in other myeloid cells. As noted above, influenza A-induced trained immunity in AMs was dependent on IFN-γ and NK cell activity (Wang et al., 2023), indicating a broader network of immunological cooperation among different innate immune cell types.

In addition to neutrophils, monocytes/macrophages, and NK cells, innate γδ T cells and innate lymphoid cells have also been shown to acquire trained immunity properties following vaccinations (Samuel et al., 2024; Taira et al., 2024; Röring et al., 2024), although their specific antitumor functions remain to be fully investigated.

### Links between trained immunity and adaptive T cells

Although trained innate immune cells exhibit potent tumor-killing capabilities, they also engage the adaptive T cell response to further enhance antitumor immunity. Dendritic cells (DCs), known for bridging innate and adaptive immunity, demonstrate enhanced antigen-presenting capabilities when stimulated with β-glucan. These DCs exhibit increased expression of primary (MHC/peptide), secondary, and tertiary signaling molecules, making them more effective at stimulating T cells within the immunosuppressive TME (Li et al., 2010). The nontoxic cholera B subunit (CTB) directly stimulates trained immunity in DCs and their antigen-presenting capability, thereby inducing potent antitumor CD8 T cell responses (Tepale-Segura et al., 2024). A combination of trained immunity inducers, such as muramyl dipeptide-containing nanoparticles and β-glucan, has shown promising results in cancer vaccine studies. In these experiments, trained groups exhibited significant reductions in tumor burden, and in some cases, complete tumor ablation. These effects were mediated by enhanced DC function, which improved the activation and infiltration of tumor-specific CD8+ T cells (Li et al., 2023a). Similarly, macrophages/monocytes trained by adenovirus or BCG vaccine also exhibited increased expression of MHC II and the costimulatory molecules CD80 and CD86 (Yao et al., 2018; Li et al., 2023b), thereby promoting T cell responses. Interestingly, macrophages trained by sepsis or the soluble algae-derived β-glucan laminarine secreted large amounts of chemokines, including CXCL16, which chemoattract tissue-resident CXCR6+ T cells, thereby promoting antitumor immunity (Broquet et al., 2024). Based on these findings, combining trained immunity with ICI therapy has been shown to induce potent antitumor immunity and reduce tumor burden, particularly in ICI-resistant tumors (Geller et al., 2022; Priem et al., 2020; Ylösmäki et al., 2021). Additionally, cytokines such as TNF-α, IL-1β, and IL-6 secreted by trained innate cells may polarize different subsets of T cells. However, their relevance to TME reprogramming remains to be fully elucidated.

### Inducers to stimulate trained immunity in cancer

Many inducers have been identified that stimulate trained immunity in the context of cancer, with relevant preclinical studies summarized in Table 1.

**Table 1.** Preclinical investigations of diverse trained immunity inducers across various tumor types.

Cancer type	Innate immune cell type	Primary stimuli	Secondary stimuli	Administration route	Ref.
Lung cancer	Alveolar macrophages	Influenza A virus	Tumor-derived factors	Intranasally (i.n.)	Wang et al., 2023
Lung cancer and melanoma	Interstitial macrophages	Yeast-derived particulate β-glucan	Tumor-derived factors e.g. MIF	Intraperitoneally (i.p.)	Ding et al., 2023
Lung cancer and melanoma	Neutrophils	β-glucan from *Trametes versicolor*	Unknown	i.p.	Kalafati et al., 2020
Pancreatic cancer	Macrophages	Yeast-derived particulate β-glucan	Lipopolysaccharide (LPS) and tumor-derived factors e.g. MIF	i.p. or orally	Geller et al., 2022; Woeste et al., 2023
Melanoma and bladder cancer	Monocytes	β-Glucan from *S. cerevisiae*	Tumor-derived factors	i.p.	Vuscan et al., 2024
Melanoma	Dendritic cells (DCs)	Cholera toxin B	Cholera toxin B	Intradermally (i.d.)	Tepale-Segura et al., 2024
Melanoma	Bone marrow	MTP10-HDL	LPS and tumor-derived factors	Intravenously (i.v.)	Priem et al., 2020
Hepatocellular carcinoma	Unknown	Bacillus Calmette-Guérin (BCG)	LPS	Subcutaneously (s.c.)	Vaziri et al., 2024
Bladder cancer	Monocytes	BCG	LPS	i.v.	Buffen et al., 2014
Multiple tumors	Splenic CD11b+ cells	KK2DP7	LPS	i.v.	Zhang et al., 2025
Lung cancer	Macrophages	Macrophage membrane-camouflaged BCG	LPS	i.v.	Zhang et al., 2024

.

MIF: macrophage migration inhibitory factor; MTP10-HDL: a new nanobiologic candidate; KK2DP7: a dendrimer-structured peptide derived from the immunomodulatory antimicrobial peptide DP7 (VQWRIRVAVIRK).

Beyond these well-established inducers, recent research in other diseases has identified novel triggers of trained immunity, although their effects in cancer need further investigation. Microbial-derived inducers, such as Hepatitis B virus (Hong et al., 2015), and the pathogen *Plasmodium falciparum* (Schrum et al., 2018) have been shown to effectively induce trained immunity. Hepatitis B virus exposure reprograms macrophages, enhancing their responsiveness to future infections, while *P. falciparum*, the causative agent of malaria, alters innate immune function, leading to an enhanced inflammatory state. In addition to microbial agents, several non-microbial inducers have emerged as potent triggers of trained immunity. Lipoproteins (van der Valk et al., 2016; Bekkering et al., 2014), such as low-density lipoprotein, influence innate immune cells and contribute to chronic inflammation. Uric acid (Crişan et al., 2017; Crisan et al., 2016), a byproduct of purine metabolism, has been implicated in inducing inflammatory responses. Hormones like aldosterone (van der Heijden et al., 2020b) and catecholamines (van der Heijden et al., 2020a) modulate immune function, linking neuroendocrine regulation to trained immunity. S100-alarmins (Ulas et al., 2017) and heme (Dutra and Bozza, 2014; Jentho et al., 2021), both released during cellular damage or stress, act as danger signals that prime innate immune cells for more robust responses to subsequent stimuli. Additionally, activators of the liver X receptor pathway (Sohrabi et al., 2020) and interferons (Kamada et al., 2018) promote enhanced antiviral defenses through trained immunity. Cytokines such as GM-CSF and IL-3 (Borriello et al., 2016), essential for hematopoiesis, also enhance immune cell responsiveness. Poly (I:C) (Yap et al., 2022), a synthetic analog of viral double-stranded RNA, and leptin (Flores Gomez et al., 2024), a hormone involved in metabolism and inflammation, contribute to trained immunity by modulating innate immune cell function. Notably, a Western diet, characterized by high intake of fats and sugars, promotes trained immunity through chronic low-grade inflammation, altering the baseline state of innate immune cells and potentially predisposing individuals to metabolic and inflammatory diseases (Christ et al., 2018; Christ and Latz, 2019).

A particularly significant advancement comes from high-throughput screening conducted by Knight et al., which identified 32 novel small molecule inducers of trained immunity, more than doubling the previously known number. Of these, seven were further validated for their ability to augment cytokine responses in both in vitro and in vivo, making substantial progress in expanding the repertoire of trained immunity inducers and highlighting their therapeutic potential (Knight et al., 2024). However, the effectiveness of these novel inducers for cancer treatment needs to be further determined.

### Trained immunity in preclinical tumor models

#### Tumor-suppressive roles of trained immunity in cancer

This section reviews the tumor-suppressive effects of trained immunity across multiple cancer types. Evidence from skin, lung, gastrointestinal, and other cancers demonstrates how metabolic and epigenetic reprogramming of innate immune cells can enhance antitumor responses, reshape the TME, and improve therapeutic outcomes. These insights underscore the potential of trained immunity as a promising strategy in cancer immunotherapy.

#### Skin cancer

Trained immunity plays a critical role in boosting antitumor responses in melanoma. Previous studies have demonstrated that the β-glucan compound ABB i16 induces trained immunity in monocytes and suppresses melanoma in mouse models (Vuscan et al., 2024). The CTB subunit enhances DC training via metabolic reprogramming, while MTP10-HDL improves ICI efficacy by reprogramming myeloid cells (Priem et al., 2020; Vuscan et al., 2024) to control melanoma progression. Electrochemotherapy and BCG therapy also activate trained immunity, reshaping the TME to support anti-melanoma immune responses (Cardillo et al., 2021; Milicevic et al., 2023). Additionally, cytokines such as GM-CSF and IL-3 may modulate trained immunity in the treatment of metastatic melanoma, although their precise roles remain to be fully elucidated (Borriello et al., 2016).

#### Lung cancer

Trained immunity enhances antitumor activity in lung cancer by reprogramming macrophage function and boosting local immune defenses. For example, yeast-derived β-glucan particles (WGP) induce sustained immune responses in lung interstitial macrophages, enhancing their phagocytic and cytotoxic activities, which in turn help reduce primary lung cancer and tumor lung metastasis (Ding et al., 2023). Virus-induced trained immunity, such as following influenza A infection, reprograms AMs to induce long-lasting pulmonary antitumor immunity (Wang et al., 2023). Furthermore, BCG vaccination has been shown to generate lung-resident memory macrophages with trained immunity properties, suggesting the potential of vaccine-induced innate memory for lung cancer prevention (Jeyanathan et al., 2022). Given the complex and heterogeneous nature of lung cancer and its interactions with the immune system, personalized and targeted therapeutic strategies are essential. Leveraging trained immunity through vaccines such as MV130 and BCG, or modulating macrophage activity via metabolic and epigenetic interventions, presents a promising avenue for enhancing antitumor immunity in lung cancer.

#### Gastrointestinal cancer

In gastric cancer, *Helicobacter (H.) pylori* infection induces a unique form of trained immunity that heightens monocyte responsiveness via NF-κB signaling, contributing to chronic inflammation and tumorigenesis (Frauenlob et al., 2023). This immune imprinting contrasts with the tolerance induced by other microbes and reflects *H. pylori*’s dual ability to stimulate and evade immune responses (Dore and Pes, 2024). In liver cancer, trained immunity shows therapeutic potential through various mechanisms. β-Glucan enhances immune protection in hepatocytes via mitophagy induction and inhibition of pyroptosis (Wang et al., 2024). BCG therapy promotes apoptosis, reduces fibrosis, and recruits immune cells in hepatocellular carcinoma (HCC) models (Vaziri et al., 2024). SQLE-derived metabolites activate LXR, enhancing macrophage-driven antitumor immunity (Liu et al., 2024). Additionally, pre-operative exercise and prior infections reprogram Kupffer cells toward anti-inflammatory phenotypes, improving liver resilience and potentially preventing tumor progression (Musrati et al., 2024; Zhang et al., 2021). In pancreatic cancer, β-glucan enhances peripheral trained immunity, particularly when combined with IRE, resulting in decreased tumor burden and improved survival (Geller et al., 2022; Woeste et al., 2023). These findings highlight trained immunity as a valuable tool for reconfiguring the TME across gastrointestinal malignancies.

#### Other cancers

In metastatic breast cancer models, trained immunity induced by particulate β-glucan has been shown to significantly prolong survival and reduce lung metastases (Ding et al., 2023). In parallel, β-glucan-induced trained immunity reprograms bone marrow-derived macrophages into an antitumor state. However, in the bone metastatic niche, elevated BMP signaling suppresses this reprogramming effect (Ihle et al., 2024). Notably, inhibition of BMP signaling prior to tumor establishment enhances the trained immunity response, restrains bone metastasis progression, and improves survival in mouse models (Ihle et al., 2024). In bladder cancer, modified BCG strains like BCG-disA-OE amplify myeloid cell reprogramming and improve therapeutic efficacy (Singh et al., 2022; Um et al., 2023). Autophagy is essential for BCG-induced epigenetic reprogramming in monocytes, with its inhibition impairing trained immunity (Buffen et al., 2014). Notably, the efficacy of BCG in treating bladder cancer is influenced by both the route of administration and different BCG strains (Xu et al., 2024). Intravenous BCG leads to greater CTL infiltration and fewer MDSCs compared to intravesical instillation (Atallah et al., 2024). Additionally, β-glucan shows complementary antitumor effects in bladder cancer (Vuscan et al., 2024). In brain cancers, BCG vaccination has been proposed as a preventive approach, though data are limited and emphasize the need for robust registry-based research in high-incidence regions (Singh et al., 2023).

#### Tumor-promoting effects of trained immunity in cancer progression

Despite its protective roles, trained immunity may paradoxically contribute to tumor development in certain contexts. Emerging evidence suggests that trained immunity can facilitate tumor progression by sustaining chronic inflammation and promoting metabolic dysregulation. In liver cancer, early hyperlipidemia and DNA hypomethylation drive metabolic reprogramming and enhance pro-inflammatory pathways, predisposing the liver to fibrosis and HCC (Drummer et al., 2021; Xu et al., 2023). Disruption of RIPK1 and related immune–metabolic pathways further links trained immunity to obesity-associated liver inflammation and potential tumorigenesis (Sohrabi and Reinecke, 2021). Oxidative stress, inflammasome activation, and interleukin-1 family cytokines also contribute to a tumor-promoting microenvironment in liver disease (Barbier et al., 2019; Dallio et al., 2021). In lung cancer, macrophage-mediated inflammation – triggered by surfactant phosphatidylcholines or viral infections – may create an immunosuppressive niche that promotes tumor growth and immune evasion (da Costa Loureiro et al., 2020; Snell et al., 2017). Elevated levels of cytokines such as IL-6, commonly seen in trained innate immune cells, further support cancer cell survival and invasion (Briukhovetska et al., 2021; Rašková et al., 2022). These findings underscore the dual-edged nature of trained immunity, wherein persistent activation may inadvertently enhance tumorigenic potential.

### Induction of trained immunity for cancer treatment in human patients

#### BCG and cancer treatment

Although the term ‘trained immunity’ has only been formally recognized in recent years, its immune-enhancing effects have long been used, intentionally or unintentionally, in cancer treatment. The profound role of trained immunity in cancer was first noted through observations of the relationship between pathogen infections and tumors. In 1893, Dr. William Coley reported the antitumor effects of bacterial infections, making the beginning of cancer treatments that harness non-specific immune responses triggered by certain pathogens or their components (Hoption Cann et al., 2003). In 1928, Pearl et al. conducted a case-by-case autopsy study of two matched cohorts comprising 1632 patients and found that individuals with active tuberculosis lesions had significantly lower incidence of cancer (6.6% vs. 16.3%) (Pearl, 1928). Later, Old et al. demonstrated the protective effect of BCG inoculation against tumors in mouse models (Old et al., 1959). Today, it is well established that the administration of BCG induces prominent trained immunity in both mice and humans (van Puffelen et al., 2020). BCG immunotherapy remains the most classic and successful clinical application of trained immunity, particularly in the treatment of bladder cancer (van Puffelen et al., 2020; Chen et al., 2023). Originally developed in 1921 as a tuberculosis vaccine from an attenuated strain of *Mycobacterium bovis* (World Health Organization, 2018), BCG was later demonstrated to exert antitumor effects in humans. In 1976, Dr. Morales et al. reported that nine patients with superficial bladder tumors who received 5 mg of BCG intradermally and 120 mg BCG via intracavitary instillation exhibited a marked reduction in tumor recurrence rate (Morales et al., 2002). This study helped establish BCG as an adjuvant therapy for non-muscle-invasive bladder cancer (Pettenati and Ingersoll, 2018). The non-specific protection conferred by the BCG vaccine has also shown promise in treating other solid tumors, including colorectal cancer (Nishida et al., 2019; Uyldegroot et al., 2005; Gray et al., 1989), melanoma (Nishida et al., 2019; Kremenovic et al., 2020), non-small-cell lung cancer (Nishida et al., 2019; Yamamura et al., 1979), hepatobiliary cancers (Nishida et al., 2019), and childhood leukemia (Morra et al., 2017). These broad protective effects are believed to be mediated by trained immunity (van Puffelen et al., 2020; Pettenati and Ingersoll, 2018), suggesting that existing vaccines with well-established safety profiles may serve as valuable platforms for developing novel antitumor immune inducers.

#### β-Glucan and cancer treatment

As mentioned earlier, β-glucan has shown profound antitumor effects in preclinical studies. Clinical trials using various forms of β-glucan have also been conducted in patients with different malignancies. In a Phase II study of patients with advanced NSCLC, the combination of β-glucan with EGFR-targeting agents and chemotherapy significantly improved ORR. Furthermore, patients with higher levels of anti-β-glucan antibody (ABA) exhibited better overall survival than those with lower ABA levels (Thomas et al., 2017). Similarly, a randomized, controlled trial showed that combining β-glucan with VEGF-targeting agents and chemotherapy modestly increased the ORR in patients with advanced NSCLC (Engel-Riedel et al., 2018). Albeituni et al., 2016 explored the immunological effects of oral WGP in NSCLC patients and found a significant reduction in MDSCs in peripheral blood following WGP administration. Oral WGP treatment also led to downregulation of the transcription factor c-Maf in circulating monocytes from NSCLC patients (Liu et al., 2020). In 50 patients with esophageal carcinoma, the addition of lentinan to chemotherapy enhanced clinical efficacy by elevating pro-inflammatory cytokine levels and lowering anti-inflammatory interleukin levels (Wang et al., 2012). Similar results were observed in breast cancer patients treated with β-glucan (Ostadrahimi et al., 2024; Ostadrahimi et al., 2014). Moreover, the combination of β-glucan with alemtuzumab and rituximab treatment led to a high complete response rate in patients with high-risk chronic lymphocytic leukemia (Zent et al., 2015). β-Glucan has also shown antitumor effects when applied locally. Laccetta et al., 2015 reported that intravaginal β-glucan spray significantly improved disease clearance rates in patients with atypical squamous cells of undetermined significance and low-grade squamous intraepithelial lesions. Clinical trials evaluating β-glucan treatment in human cancer patients are summarized in Table 2.

**Table 2.** Summary of β-glucan treatment effects in various tumor types.

Trial type	Tumor type	β-Glucan form/ administration route	Treatment arm	Control arm	Main effects of β-glucan	Ref.
RCT Phase II	High-risk relapsed metastatic neuroblastoma	A gel formulation /P.O.	Ganglioside vaccine+β-glucan	Ganglioside vaccine	Enhanced seroconversion of anti-ganglioside IgG1	Cheung et al., 2023
RCT	Breast cancer	Soluble/P.O.	Chemo.+β-glucan	Chemo.+placebo	Decreased IL-4; increased IL-12; enhanced energy intake	Ostadrahimi et al., 2014
RCT	Breast cancer	Soluble/P.O.	Chemo.+*Lactobacillus rhamnosus* strain Heriz I+β-glucan	Chemo.+placebo	Decreased IL-4	Ostadrahimi et al., 2024
SACT	Advanced breast cancer	Soluble/P.O.	β-Glucan	-	Increased peripheral monocyte count	Demir et al., 2007
SACT	NSCLC	Particulate/P.O.	β-Glucan	-	Decreased MDSC	Albeituni et al., 2016
RCT Phase II	Advanced NSCLC	Soluble/I.V.	Chemo.+Cetuximab+β-glucan	Chemo.+Cetuximab	Increased ORR	Thomas et al., 2017
RCT	Advanced NSCLC	Soluble/I.V.	Chemo.+bevacizumab+β-glucan	Chemo.+bevacizumab	Relatively increased ORR	Liu et al., 2020
CCT	Esophageal carcinoma	Soluble/I.V.	Chemo.+lentinan	Chemo.	Increased chemotherapeutic efficacy and pro-inflammatory ILs, decreased anti-inflammatory ILs	Wang et al., 2012
SACT	Gastric cancer	Soluble/I.V.	Chemo.+lentinan	-	Increased quality-of-life scores	Kataoka et al., 2009
SACT	Pancreatic cancer	Superfine dispersed/P.O.	Superfine dispersed lentinan	-	Increased quality-of-life scores	Kataoka et al., 2009
SACT Phase II	Stage IV KRAS-mutant colorectal cancer	Soluble/I.V.	Cetuximab+β-glucan	Cetuximab	Compelling clinical activity	Segal et al., 2016
RCT	ASCUS/LSIL	Soluble/TOP.	β-Glucan	No treatment	Increased disease clearance rates	Laccetta et al., 2015
SACT Phase I/II	High-risk chronic lymphocytic leukemia	Soluble/I.V.	Alemtuzumab+rituximab+β-glucan	-	A high complete response rate	Zent et al., 2015
RCT	Mixed	NS/P.O.	Hypercaloric diet enriched in β-glucan	Hypercaloric diet	Increased energy intake	Milla et al., 2024
SACT Phase I/II	Mixed advanced	Soluble/P.O.	Chemo.+β-glucan	-	Relatively increased blood cell counts	Weitberg, 2008

RCT: randomized clinical trial; CCT: controlled clinical trial; SACT: single arm clinical trial; NSCLC: non-small cell lung cancer; ASCUS: atypical squamous cells of undetermined significance; LSIL: low-grade squamous intraepithelial lesions; NS: not specified; P.O.: Per os; I.V.: intravenous; TOP.: topical; Chemo.: chemotherapy; IL: interleukin; MDSC: myeloid-derived suppressor cells; ORR: objective response rates.

Beyond its antitumor effects, β-glucan also promotes myelopoiesis and hematopoiesis in cancer patients. In advanced breast cancer, a 15-day β-glucan treatment significantly increased peripheral monocyte counts, accompanied by elevated expression of CD95 and CD45RA on CD14-positive monocytes (Demir et al., 2007). In a cohort of patients with 11 different types of malignancies, white blood cell counts increased to varying degrees following β-glucan administration during chemotherapy (Weitberg, 2008). This effect resembles the central trained immunity induced by β-glucan in mice, where enhanced myelopoiesis is a hallmark (Mitroulis et al., 2018). Interestingly, β-glucan has also been shown to enhance the nutritional status and quality of life in cancer patients. Milla et al. reported that oral nutritional supplements enriched with β-glucan improved energy intake, particularly in patients with severe malnutrition or advanced tumor stages (Milla et al., 2024). Similarly, patients with gastric or pancreatic cancers experienced significant improvements in quality of life during chemotherapy with the addition of lentinan (Shimizu et al., 2009; Kataoka et al., 2009).

However, despite the promising antitumor effects observed in preclinical studies, the impact of β-glucan on patient survival in most clinical trials has been limited. This discrepancy highlights the importance of optimizing β-glucan formulations, administration routes, and treatment timing to enhance its clinical efficacy. Further research is needed to develop novel drug delivery systems and refine therapeutic strategies to replicate the pronounced antitumor effects seen in animal models in human patients.

#### Prospectives

**Figure 3.**
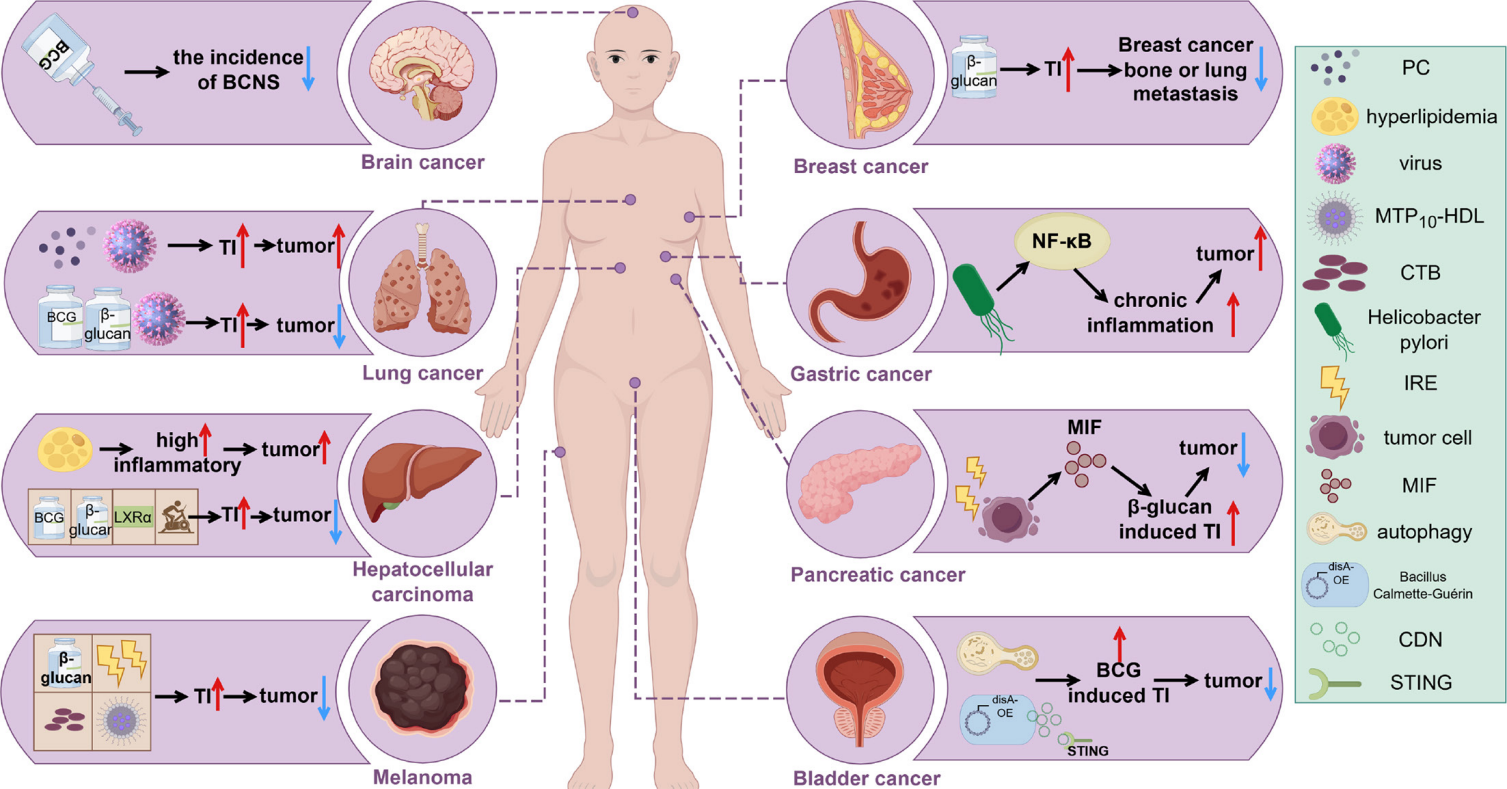
Induction of trained immunity in different tumors. The tumor-promoting or tumor-suppressing roles of various trained immunity inducers, such as BCG and β-glucan, across different cancer types are illustrated. An upward red arrow indicates an enhanced effect or tumor-promoting activity, while a downward blue arrow denotes inhibition of tumor progression. Abbreviations: BCNS, brain and central nervous system tumors; CDN, cyclic di-nucleotide; CTB, cholera toxin B; IRE, irreversible electroporation; MIF, macrophage migration inhibitory factor; MTP10-HDL, a new nanobiologic candidate; PC, phosphatidylcholine; TI, trained immunity. This figure was created using FigDraw.

Despite significant progress in the field of trained immunity and cancer (Figure 3), many challenges remain in fully harnessing this new approach for cancer immunotherapy. A critical area for future investigation involves leveraging the mechanisms of trained immunity to develop innovative cancer therapies and vaccines that integrate both classical adaptive immune memory and trained immunity. As myeloid cells are the predominant immune cells infiltrating tumors and shaping an immunosuppressive microenvironment in many solid tumors, inducing trained immunity may convert a ‘cold’ into a ‘hot’ tumor, thereby augmenting the efficacy of adaptive T cell-based immunotherapies such as ICI. In addition, adjuvants with trained immunity-inducing properties can be combined with cancer vaccines to induce long-lasting antitumor immune responses. Given the crucial role of epigenetic alterations in trained immunity (Saeed et al., 2014; van der Heijden et al., 2018), preventing epigenetic deactivation through the use of agents like DNA methyltransferase and histone deacetylase inhibitors may prolong its effect and promote durable antitumor responses. However, strategies to mitigate off-target effects and potential detrimental consequences must be explored. This is especially important as sustained inflammation can also promote tumor progression. Therefore, both the intensity and duration of trained immunity must be carefully considered when applied to cancer immunotherapy. It is also notable that different routes of administration lead to varying potencies of trained immunity. For example, intravenous BCG injection elicits stronger trained immunity than other delivery routes. This consideration is particularly relevant for β-glucan-induced trained immunity. Currently, i.p. injection of β-glucan is considered the gold standard in preclinical studies, as it induces robust trained immunity in mice. In contrast, oral delivery appears to induce a weaker response and typically requires daily administration. The potency of trained immunity is also influenced by the formulation of β-glucan (soluble vs. particulate forms) and the source of β-glucans (yeast-derived vs. bacterial or mushroom-derived).

As new trained immunity inducers continue to emerge, clear selection criteria must be established. It is important to determine whether an agent induces both central and peripheral trained immunity or exerts localized effects, such as those achieved through intratumoral injection. Furthermore, the development of personalized cancer vaccines tailored to deliver trained immunity agents to specific target cells is warranted, given that various innate cells can exhibit trained immunity features. Ideally, these strategies should activate all trained innate immune cells with antitumor potential while minimizing adverse effects. Ultimately, a deeper understanding of the mechanisms of action for various trained immunity inducers and their downstream effects on antitumor immunity is essential for advancing this therapeutic approach.

The identification of biomarkers indicative of effective trained immunity induction and their integration into future clinical investigations will be critical for selecting patients most likely to benefit from this therapy. Moorlag et al. recently conducted a study involving 323 healthy participants, collecting blood samples on day 0, as well as on days 14 and 90 post BCG vaccination, followed by comprehensive multi-omics analyses. The results indicate that chromatin profiling and functional assays can effectively identify individuals in a dormant innate immune state, enabling targeted immune-boosting interventions (Moorlag et al., 2024). Ongoing research is essential to uncover additional biomarkers that support personalized treatment strategies, including the identification of individuals most responsive to specific trained immunity stimulants, optimal dosing strategies, and biomarkers that signal waning immunity, indicating the need for re-administration. Similarly, determining the optimal timing and sequencing of trained immunity induction in combination with existing immunotherapy, such as ICI therapy, is imperative. As the induction of trained immunity requires training and elicitation, its application in preventing cancer recurrence and metastasis appears to be particularly promising. However, further clinical investigations are necessary to fully explore the potential of trained immunity in cancer immunotherapy.
